# Combined tracheoesophageal transection following a life-threatening clothesline-type blunt neck trauma: A case report

**DOI:** 10.1016/j.ijscr.2023.109173

**Published:** 2023-12-19

**Authors:** Ramin Ebrahimian, Maziar Moayerifar, Mahboobeh Gholipour, Maede Mohammadian, Mani Moayerifar

**Affiliations:** a Faculty of Medicine, Guilan University of Medical Sciences, Rasht, Iran; b Department of Vascular Surgery, Razi Hospital, Guilan University of Medical Sciences, Rasht, Iran; c School of Medicine, Guilan University of Medical Sciences, Rasht, Iran; d Razi Clinical Research Development Unit, Guilan University of medical Sciences, Rasht, Iran

**Keywords:** Tracheal transection, Esophageal injury, Clothesline-type trauma, Case report

## Abstract

**Introduction:**

Blunt neck trauma is an uncommon, life-threatening injury that may result in tracheoesophageal transection. The manifestations of these traumas are rather vague and nonspecific; therefore, the injury may be missed, if a careful attention is not paid.

**Case presentation:**

A 23-year-old young man presented with complete transection of the trachea and concurrent esophageal injury, caused by clothesline-type blunt neck trauma, while riding a motorcycle. On early examination, the patient was hemodynamically stable; however, after a few minutes, he manifested respiratory distress and progressive subcutaneous emphysema. The airway immediately was secured by inserting an endotracheal tube in distal part of the transected trachea. Afterward, the patient underwent primary repair of transected trachea and esophagus, and tracheostomy. The post-operative period was uneventful.

**Discussion:**

The blunt traumas to neck, which lead to complete transection of the trachea and the esophagus, are rare injuries. Clothesline-type injuries are the principal reasons for cricotracheal separation and further esophageal injuries. In most cases, subcutaneous emphysema is a sign of significant trauma to the aerodigestive tract. After securing the patient's airway, early surgical repair of the transected trachea and esophagus reduces the risk of further complications.

**Conclusion:**

This report discusses a rare, life-threatening presentation of blunt neck trauma called clothesline-type injury, that led to complete transection of the trachea and concurrent esophageal rupture. Establishing a secure airway for those patients with tracheal injuries is required. Repairing the injured trachea and esophagus primarily at the earliest possible time can improve the patient prognosis and prevent further complications.

## Highlights

• Blunt neck trauma is an uncommon, life-threatening injury that may lead to tracheoesophageal transection
• Progressive subcutaneous emphysema is a prominent sign, that indicates significant trauma to the aerodigestive tract
• Establishing a secure airway in patient with blunt trauma to neck would be life-saving
• Repairing the injured trachea and esophagus primarily at the earliest possible time can improve the patient prognosis and prevent further complications

## 1 Introduction

Blunt neck trauma is an uncommon, life-threatening injury that may lead to tracheoesophageal transection [1]. As the signs of the injury may be vogue and nonspecific, close attention is required not to miss any aerodigestive injuries [2]. Progressive subcutaneous emphysema is a prominent sign that indicates significant trauma to the aerodigestive tract. For all patients with tracheobronchial injuries, establishing a secure airway would be life-saving [3].

In this study, we present a young man with a clothesline-type blunt neck injury with combined tracheoesophageal transection, manifesting progressive subcutaneous emphysema and respiratory distress. This work has been reported in line with the updated consensus Surgical Case Report (SCARE) 2020 guidelines [4].

## 2 Case presentation

A 23-year-old young man was taken to the ED (Emergency Department) of the local hospital via EMS (Emergency Medical Services) with a clothesline-type injury to the anterior neck caused by a rope, while riding a motorcycle. The patient was hemodynamically stable and conscious at the scene. He was provided with 100 % Oxygen via face mask, and his cervical spine was immobilized using a hard collar. There wasn't any open wound seen at the site of injury.

**Fig. 1:**
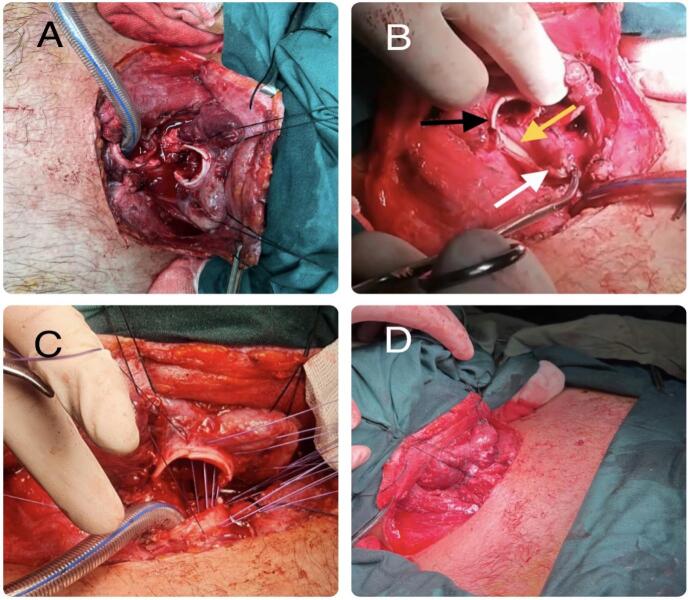
Intraoperative photograph of surgical field: (A) Complete transected trachea (B) Black arrow indicating transected trachea, white arrow indicating ruptured esophagus, and yellow arrow indicating NG tube inserted in the esophagus (C) Approximation two ends of the transected trachea using interrupted absorbable suture (D) After primary repair of the transected trachea. (For interpretation of the references to color in this figure legend, the reader is referred to the web version of this article.)

**Fig. 2:**
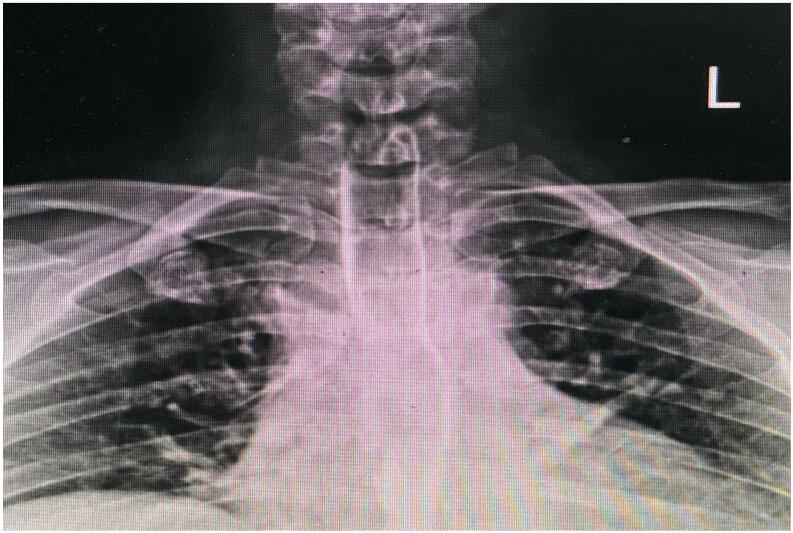
One week post-operative esophagography: No signs of aspiration or leakage are noticed.

At ED vital sign included a blood pressure of 110/70 mm Hg, a pulse rate of 50 beats/min, a respiratory rate of 32 breaths/min, and an axillary temperature of 37.4 °C, and the pulse oximetry oxygen saturation was 82 %. A few minutes after arrival, the patient developed respiratory distress. Since the respiratory sounds were absent at the left hemithorax, a chest tube was inserted by the center's general surgeon. But the respiratory distress did not vanish and the oxygen saturation did not increase. On further examination, subcutaneous emphysema was noticed in the patient's neck. After a failed trial of orotracheal intubation, the center's surgeon decided to explore the injury site, as he was suspicious of tracheal injury. Opening the site of injury with a transverse incision, a complete transected trachea was noticed at the subglottic area. Blood vessels were intact, and no sign of active bleeding was present. A 7.5 mm endotracheal tube was inserted in the distal part of the transected trachea. After providing a secure airway and completing the Primary survey, on Secondary survey, orthopedic and neurological injuries were ruled out, and the patient was referred to Tertiary Care Trauma Hospital for further investigations. After arriving at the second center, the patient was transferred to the operating room immediately. The neck opened by a cervical collar incision extended bilaterally to SCM (Sternocleidomstoid) muscles. In addition to the complete transection of the trachea, 80 % of the esophagus circumference was ruptured. As a vascular surgeon was present throughout the surgery, any vascular injuries were ruled out. An NG tube was inserted in the esophagus, and it was repaired with 3-0 absorbable interrupted suture (Fig. 1), primarily in one layer. To prevent further tracheoesophageal fistula, an SCM muscle flap was placed between the repaired esophagus and the trachea. Afterward, the transected trachea was repaired by end-to-end anastomosis using an interrupted simple 3-0 absorbable suture. Two drains were inserted. The injury to recurrent laryngeals could not be ruled out; therefore, tracheostomy was performed two tracheal ring below the injury site, and then it was connected to a ventilator. To maintain the patient's feeding, a Jejunostomy feeding tube was inserted subsequently. Following the operation, the patient was stable and was transferred to the ICU (Intensive Care Unit). On second post-operative day, the patient was weaned from the ventilator. No drop in oxygen saturation was noticed, and he had spontaneous breathing afterward. On the Fourth post-operative day, the chest tube was removed, and on the fifth post-operative day, a fibreoptic bronchoscopy was performed. The vocal cords were seen without abnormalities, but the tracheostomy tube was not removed till further examination to prove recurrent laryngeal nerves were intact. On seventh post-operative day, drains were removed as the output was insignificant, and an esophagography was performed to study esophagus anastomosis. As no leakage or aspiration was found, the patient went on oral feeding (Fig. 2). After three days of tolerating oral feeding, the Jejunostomy feeding tube was removed. Four weeks after operation, a fibreoptic bronchoscopy was carried out. No signs of tracheal stenosis and no abnormalities in vocal cords were detected; therefore, the tracheostomy was removed.

On the fourth month follow up, the patient underwent a flexible fibreoptic bronchoscopy, which was completely normal.

## 3 Discussion

Although the neck is a mobile region of the human body, it is vulnerable to injuries. The neck is an enclosed space containing many vital structures [1]. Blunt trauma accounts for only 5 % of all the neck traumas [5]. Those blunt traumas to neck, which lead to complete transection of the trachea and the esophagus, are rare injuries, that occur in less than 1 % of trauma cases in major centers. As these injuries are fatal and life-threatening, most patients cannot make it to hospital and pass away in the field [[6], [7], [8]]. In most cases, subcutaneous emphysema is a sign of significant trauma to the aerodigestive tract. Patients with tracheal transection often present with massive mediastinal and deep cervical emphysema without pneumothorax [5]. Clothesline types of injuries are a category of blunt neck trauma, that occur when someone strikes a horizontal rope or cable, while riding a motorcycle, waterskiing, and while running. These types of blunt injuries are the principal reasons for cricotracheal separation and further recurrent laryngeal or esophageal injuries, and need to be managed instantly to prevent respiratory distress [[9], [10], [11]].

Disruption of the trachea in blunt neck trauma such as clothesline types injuries, result from the shearing force caused by rapid deceleration [3]. Manifestations of tracheal transection subsequent to non-penetrating trauma are nonspecific [12]; moreover, intact peritracheal tissue sustains airway column, so it would be difficult to discover tracheal transection in patients with blunt trauma to the neck [2,3,13].

After assuring a secure airway and maintaining the patient's oxygenation, the patient should undergo a complete trauma assessment to examine the intensity of airway injury and assess any simultaneous injuries to other organs. The most common organ that can be injured in tracheobronchial injuries is the esophagus, and there is also a probability of up to 60 % concurrent recurrent laryngeal nerve injury, particularly when the cricoid cartilage is fractured and the trachea is transected [6,7,9].

For patients with blunt trauma to the neck that manifest imminent airway obstruction or “red flags” such as stridor and respiratory distress, definitive airway management is mandatory. Other patients that are hemodynamically stable and develop none of the red flags can undergo fibreoptic bronchoscopy examination [14].

To maintain oxygenation, different methods can be performed, but all of them carry their threats when applied to a patient with laryngotracheal injuries [14]. Positive pressure ventilation via a face mask may lead to air leak through the site of laryngotracheal rupture. It can cause progressive emphysema, as happened to our patient in this case. Crycothyroidotomy is not recommended in case of laryngotracheal rupture, as it can deteriorate the damaged anatomy. Blind intubation may transform a partial transection to a complete transection, or can displace fractured cartilage, or the tube may go out of the trachea via traumatic defect and establish a false passage [3,15].

To ensure a secure airway for the patient with a complete transected trachea, an endotracheal tube is inserted into the distal part of the transected trachea, using a transverse incision. In our case, the patient's airway had been stabilized using this method [6]. Afterwards, the trachea should be repaired primarily by end-to-end interrupted simple suture [2]. Early surgical repair reduces the risk of subglottic stenosis and ameliorates the airway, in addition to voice outcomes [16]. We use a 3–0 one-layer absorbable interrupted suture with an interval distance of 3–4 mm with caution to the trachea blood supply, to prevent subsequent complications such as a stricture. Esophageal injuries could be managed conservatively, or by primary repair, based on the site of injury and the extent of laceration. As oral intake is forbidden in these patients, the nutrition needs to be maintained by a Jejunostomy feeding tube or parenterally [7]. A flap of muscle, parietal pleura, or pericardium, should be placed between the repaired esophagus and trachea to prevent the development of delayed tracheoesophageal fistula [6,7]. In our case. a flap of the SCM muscle was placed between the repaired esophagus and the trachea.

The prognosis of patients improves by a prompt diagnosis of injured airway, and performing immediate airway management and neck exploration [17].

## 4 Conclusion

This report discusses a rare, life-threatening presentation of blunt neck trauma called clothesline-type injury, that led to complete transection of the trachea and concurrent esophageal rupture. Careful examination is mandatory to ensure the sufficiency of the patient's airway in the primary survey. Establishing a secure airway for those patients with tracheal injuries is required. Subsequent to primary and secondary survey a complete head and neck examination is necessary to figure out any additional injuries. Repairing the injured trachea and esophagus primarily at the earliest possible time can improve the patient prognosis and prevent further complications.

## Ethical approval

not required for case reports at our hospital. Single case reports are exempt from ethical approval in our institutions.

## Funding

The research received no external funding.

## Author contribution

**Ramin Ebrahimian:** Surgeon, Supervision, Conceptualization, Visualization and Writing-review & editing. **Maziar Moayerifar:** Surgeon, Conceptualization, Visualization, Writing-original draft and Writing-review & editing. **Mahboobeh Gholipour:** Conceptualization, Visualization, Writing-original draft and Writing-review & editing. **Maede Mohammadian:** Writing-original draft and Writing-review & editing. **Mani Moayerifar:** Surgeon, Conceptualization, Visualization, Writing-original draft and Writing-review & editing.

## Guarantor

The guarantor in this study is Ramin Ebrahimian.

## Registration of research studies

No applicable-single case report.

## Consent

Written informed consent was obtained from the patient for publication of this case report and accompanying images. A copy of the written consent is available for review by the Editor-in-Chief of this journal on request.

## Declaration of competing interest

There are none to declare.
